# Supplementary material for: Upregulated Hexokinase-2 in Airway Epithelium Regulates Apoptosis and Drives Inflammation in Asthma via Peptidylprolyl Isomerase F
Source: Cells. 2025 Jul 1;14(13):1004. doi: 10.3390/cells14131004 (PMC12248590; doi:10.3390/cells14131004)
Supplement: Supplementary file 1 [file cells-14-01004-s001.zip › cells-3671480-supplymental image.pdf]

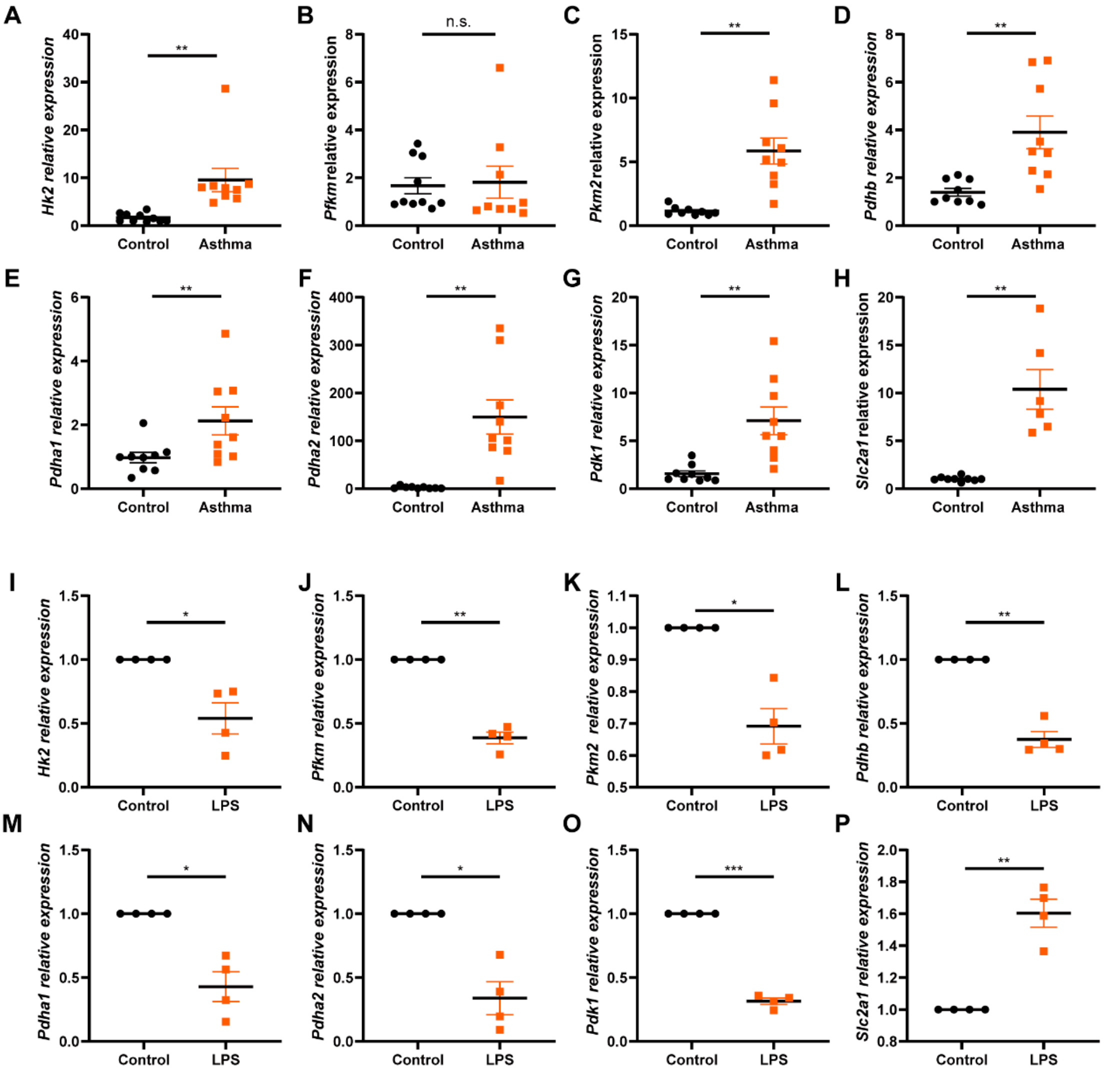

Figure S1. The key enzymes expression involved in glucose metabolism altered in mice of asthma models (**A**-**H**) RT-PCR analysis of the key enzymes expression involved in glucose metabolism: HK2 (**A**), PFKM (**B**), PKM2 (**C**), PDHB (**D**), PDHA1 (**E**), PDHA2 (**F**), PDK1 (**G**), and SLC2A1 (**H**) in mice of asthma models induced by OVA/Alum. Each group included 6-9 samples. (**I**-**P**) RT-PCR analysis of the key enzymes expression involved in glucose metabolism: HK2 (**I**), PFKM (**J**), PKM2 (**K**), PDHB (**L**), PDHA1 (**M**), PDHA2 (**N**), PDK1 (**O**), and SLC2A1 (**P**) in mice of asthma models induced by OVA/Alum. Each group included 6-9 samples. The data were shown as mean ± SEM, statistical differences were determined with a student's t test for comparison between the two groups. *$P < 0.05$, ** $P < 0.01$, *** $P < 0.001$, ns = not significant.

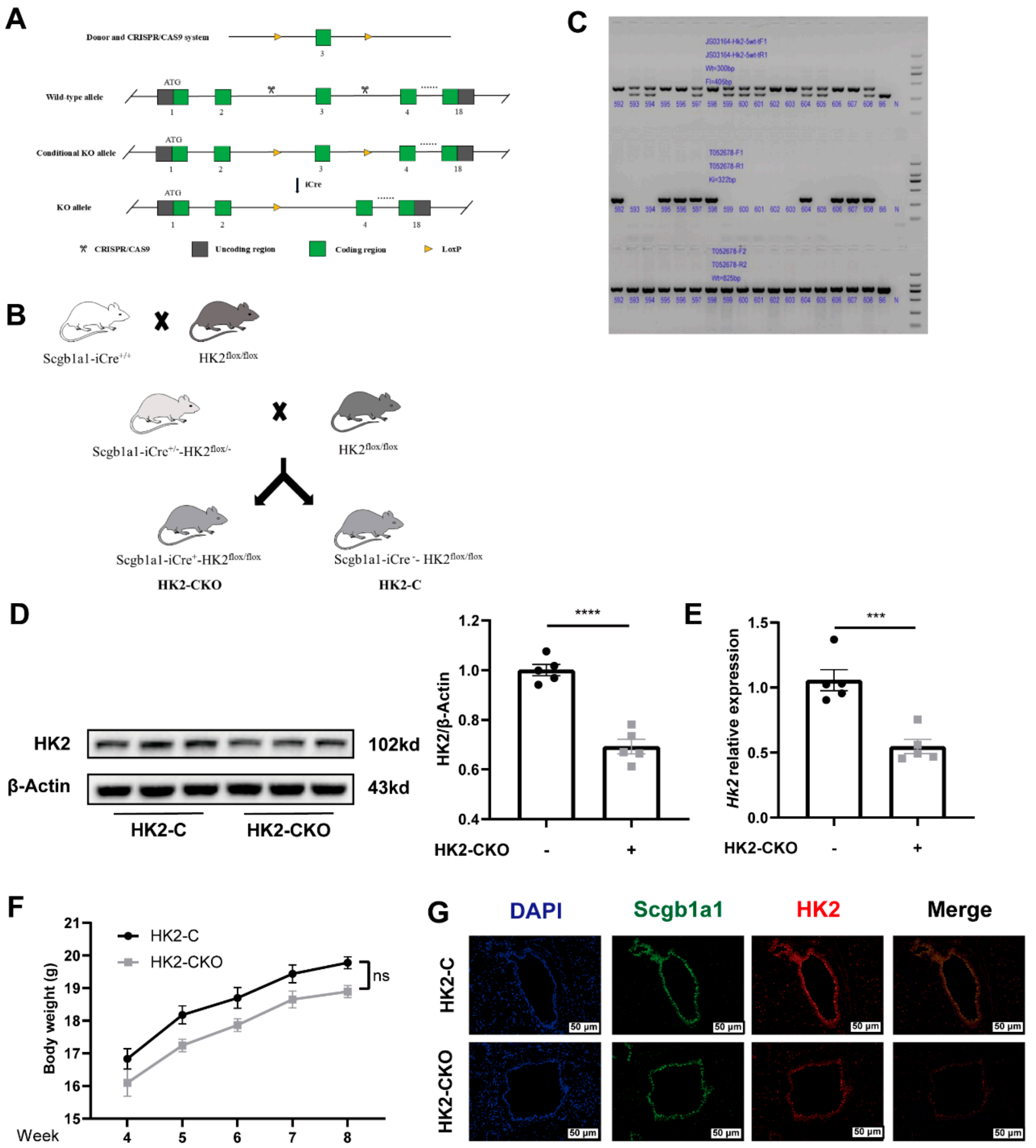

Figure S2. Generation of club-cells specific knockout mice. (**A-B**) construction strategy and breeding process of HK2-C and HK2-CKO mice. (**C**)Southern blot to analysis the flox allele form HK2$^{flox/flox}$, WT and HK2flox/- and the Scgb1a1-iCre allele. (**D**) Western blot analysis of HK2 expression in the lung of HK2-C and HK2-CKO mice. (**E**) RT-PCR analysis of HK2 expression in the lung of HK2-C and HK2-CKO mice. (**F**) The weight change of HK2-C and HK2-CKO mice for four weeks. The data were shown as mean ± SEM by two-way ANOVA. ns = no significance. (**G**) Representative co-immunostainings of HK2 and Scgb1a1 in HK2-CKO mice and HK2-C mice.

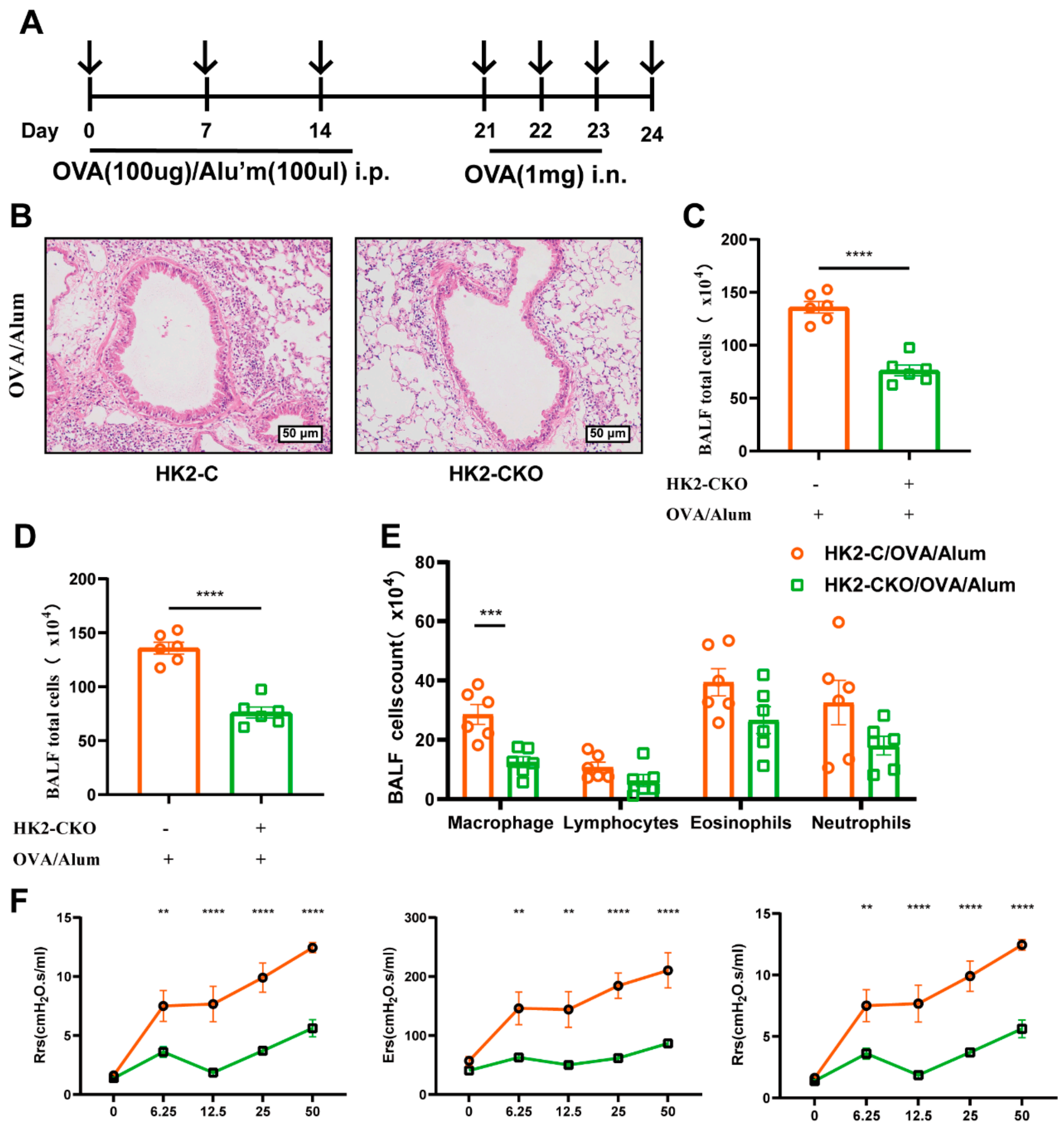

Figure S3. HK2 silencing in the airway epithelium also prevents against asthma induced by OVA/Alum. (**A**) Experimental scheme outlining the procedures. (**B**-**C**) Images and statistical graph (n = 6) of lung histology of HK2-CKO and HK2-C mice following OVA/Alum induction (stained with HE. The images were taken under original magnification ×200. Scale bar, 50 μm. (**D**) Total cells. (**E**) Differential counts of inflammatory cells in BALF of HK2-CKO mice and HK2-C mice after OVA/Alum induction (n = 6). (**F**) AHR, including Rrs, Errs, and Crs, was recorded 24 hours after the last challenge. (n = 6). The data were shown as mean ± SEM by t-students test. **** p <0.0001, ** p <0.01, and * p < 0.05. n.s.= no significance.

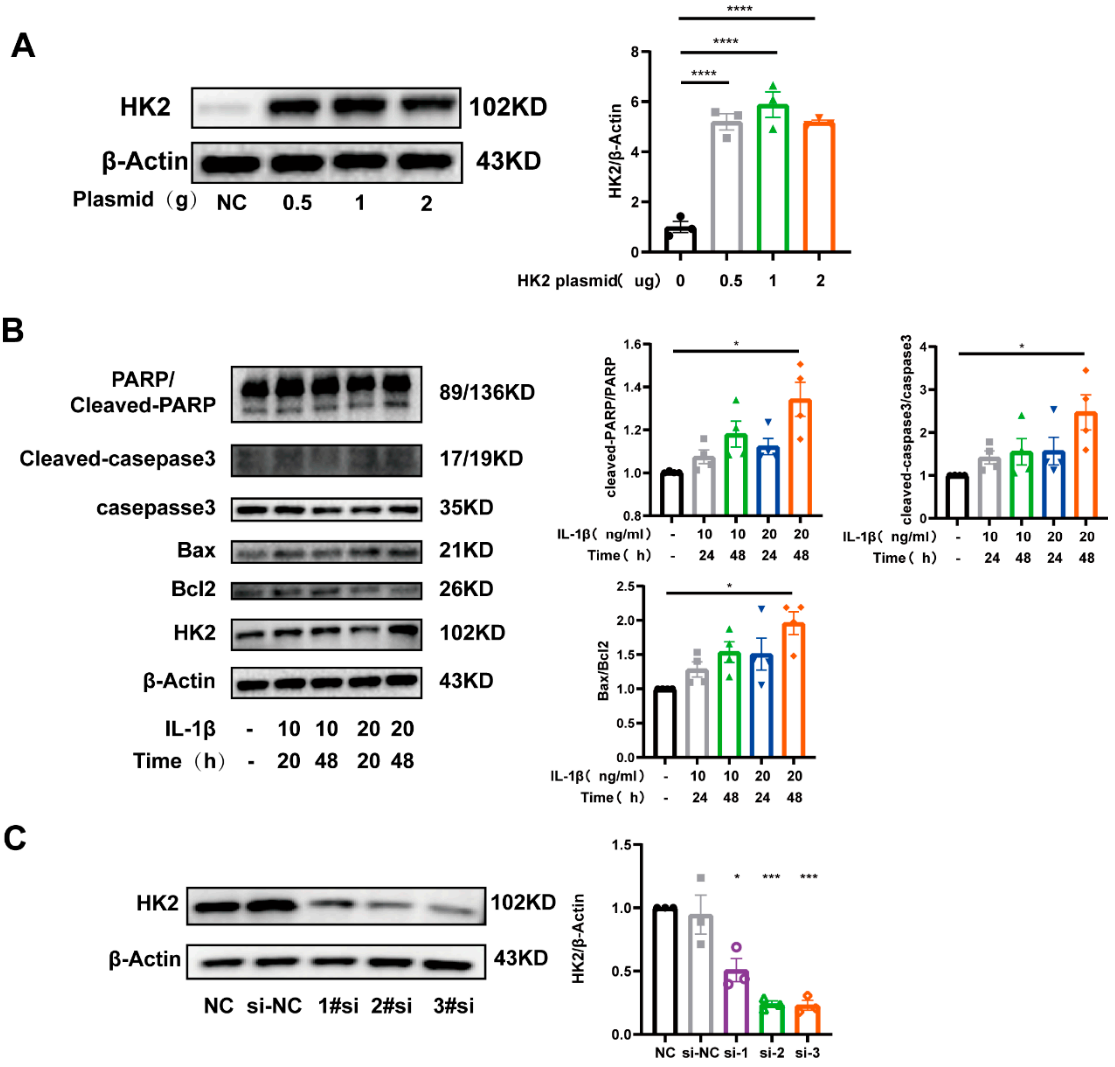

Figure S4. (**A**) Western blots analysis of HK2 protein expression in Beas-2b cells transfected with HK2 plasmid or control plasmid (n=3 per group). (**B**) Western blot analysis HK2, PARP1, cleaved PARP1, caspase-3, cleaved caspase-3, Bax, and Bcl-2 induced by IL-1β. (**C**)Western blots analysis of HK2 protein expression in Beas-2b cells transfected with HK2 si-RNA or control si-RNA (n=3 per group)

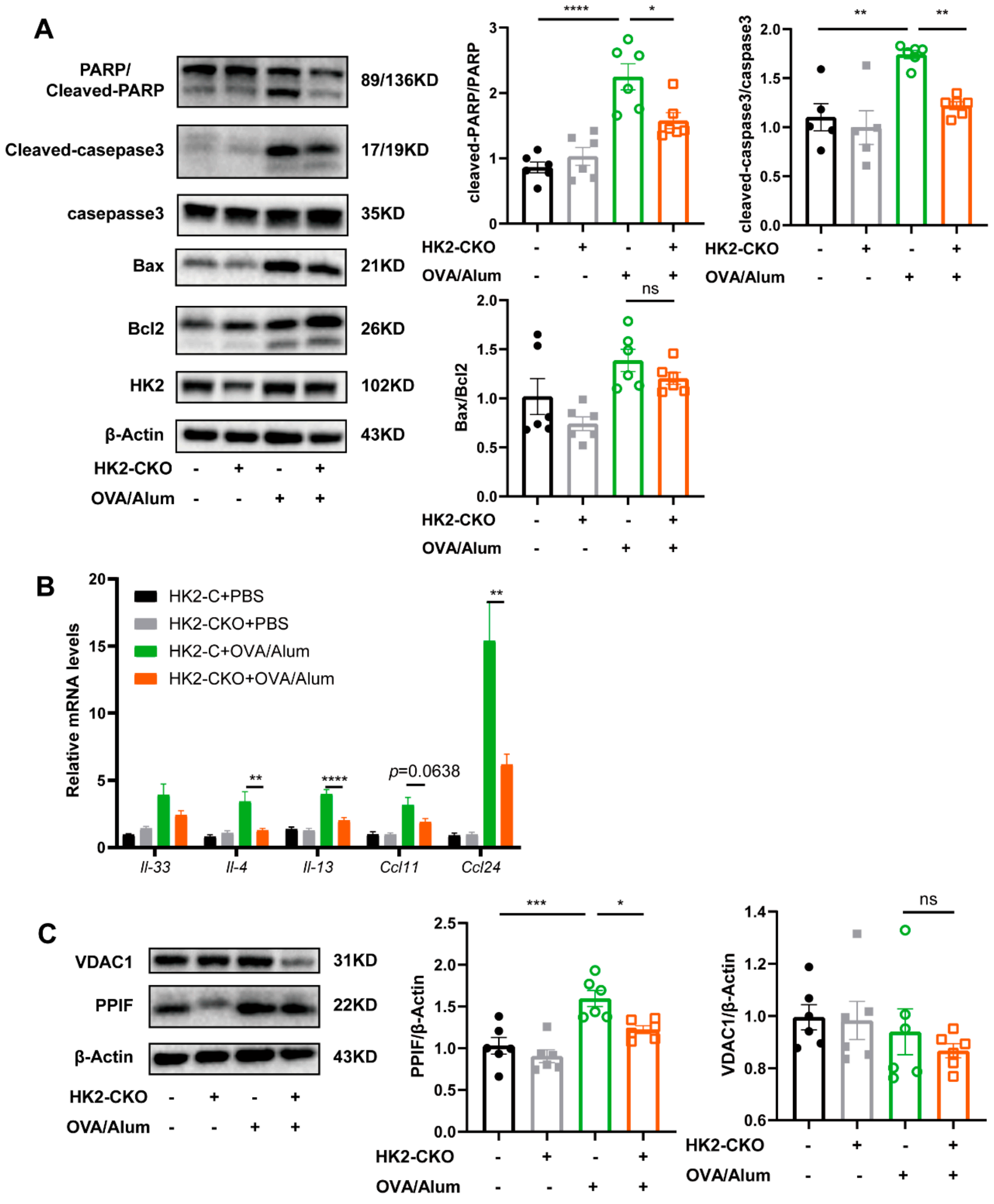

Figure S5. HK2 regulated cell death and inflammatory reaction in asthmatic mice induced by OVA/Alum. (**A**) Western blot analysis of HK2, PARP1, cleaved PARP1, caspase-3, cleaved caspase-3, Bax, and Bcl-2 in lungs of OVA/ Alum -immunized HK2-C and HK2-CKO mice (n=6 per group) (**B**) mRNA levels of IL-33, IL-4, IL-13, CCL11and CCL24 in lungs of OVA/ Alum -immunized HK2-C and HK2-CKO mice (n=6 per group) (**C**) Western blot analysis of VDAC1 and PPIF protein expression in vivo. The data were shown as mean ± SEM by one-way ANOVA. * p <0.05, ** p <0.01, *** p <0.001, **** p <0.0001 ns = no significance
