# Supplementary material for: Upregulated Hexokinase-2 in Airway Epithelium Regulates Apoptosis and Drives Inflammation in Asthma via Peptidylprolyl Isomerase F
Source: Cells. 2025 Jul 1;14(13):1004. doi: 10.3390/cells14131004 (PMC12248590; doi:10.3390/cells14131004)
Supplement: Supplementary file 1 [file cells-14-01004-s001.zip › cells-3671480-supplymental table.pdf]

Supplemental Table

Supplemental Table S1. Fundamental characteristics of subjects

Characteristics	Asthma Patients	Healthy Subjects	P-value
Number	24	20	
Sex (F/M)	15/9	11/9	0.760
Age (y)	41.33 (29.00–52.25)	37.75 (25.00–53.75)	0.079
BMI (kg/m2)	24.10 ± 4.65	22.95 ± 3.23	0.332
ACT	16 (14–17)	NA	
ACQ	1.79 (1.49–2.08)	NA	
Lung function			
FEV1 (L)	2.74 ± 0.77	3.20 ± 0.91	0.075
FVC (L)	93.02 ± 11.65	100.35 ± 10.10	0.033*
FVC % predicted	3.75 (3.05–4.43)	3.77 (2.85–4.52)	0.953
FEV1% predicted	108.20 ± 11.65	99.60 ± 9.85	0.012*
FEV1/FVC (%)	73.26 ± 9.38	85.41 ± 5.37	< 0.0001*
Methacholine PD20 (mg)	0.3246 (0.0275–0.5291)	NA	
FENO	86 (54–118)	NA	
IgE (IU/mL)	337.18 (120.70–302.40)	35.32 (19.89–49.53)	0.0001*
Blood eosinophils (%)	5.65 ± 3.40	1.50 ± 0.81	< 0.0001*
Sputum eosinophils (%)	18.39 (9.02–27.76)	NA	
Blood neutrophils (%)	52.95 ± 9.89	55.64 ± 7.17	0.326
Sputum neutrophils (%)	50.79 (40.53–61.04)	NA	

Values are presented as mean SD or median (interquartile range); BMI, body mass index; ACT, Asthma Control Test; ACQ, Asthma Control Questionnaire; NA, not applicable; PD20, provocation dose resulting in 20% fall in baseline FEV1; FENO, fraction of exhaled nitric oxide. *$p < 0.05$ vs Healthy Subjects.

Supplemental Table S2. The primers for RT-qPCR.

mouse IL-6 forward	CTGCAAGAGACTTCCATCCAG
mouse IL-6 reverse	AGTGGTATAGACAGGTCTGTTGG
mouse KC forward	TGCGAAAAGAAGTGCAGAGA
mouse KC reverse	TACAAACACAGCCTCCCACA
mouse Il10 forward	GCTGGACAACATACTGCTAACC
mouse Il10 reverse	CCCAAGTAACCCTTAAAGTCCTG
mouse CCL20 forward	AAGACAGATGGCCGATGAAG
mouse CCL20 reverse	AGCCCTTTTCACCCAGTTCT
mouse IL4 forward	GGCATTTTGAACGAGGTCAC
mouse IL4 reverse	AAATATGCGAAGCACCTTGG
mouse IL13 forward	GCAACATCACACAAGACCAGA
mouse IL13 reverse	GTCAGGGAATCCAGGGCTAC
mouse CCL11 forward	GAATCACCAACAACAGATGCAC
mouse CCL11 reverse	ATCCTGGACCCACTTCTTCTT
mouse CCL24 forward	AGGCAGTGAGAACCAAGT
mouse CCL24 forward	GCGTCAATACCTATGTCCAA
mouse -IL-33-F	TGCTGCGTCTGTTGACACATTGAG
mouse -IL-33-R	CATCCACACCGTCGCCTGATTG
mouse β-Act forward	GGCTGTATTCCCCTCCATCG
mouse β-Act reverse	CCAGTTGGTAACAAATGCCATGT
human IL-6 forward	TGAAAGCAGCAAAGAGGCACT
human IL-6 reverse	GCAAGTCTCCTCATTGAATCCAG
human IL-8 forward	CGCCTTTACAATAATTTCTGTGTTGGCG

human IL-8 reverse	CTTGGCAGCCTTCCTGA TTTCT
human CCL20 forward	CGAATCAGAAGCAGCAAGCAA
human CCL20 reverse	TTGCGCACACAGACAACTTT
human 18s forward	GTAACCCGTTGAACCCCATT
human 18s reverse	CCATCCAATCGGTAGTAGCG
